# Screening and Identification of Putative Long Non-Coding RNA in Childhood Obesity: Evaluation of Their Transcriptional Levels

**DOI:** 10.3390/biomedicines10030529

**Published:** 2022-02-23

**Authors:** Manuela Cabiati, Martina Fontanini, Manuel Giacomarra, Gianfranco Politano, Emioli Randazzo, Diego Peroni, Giovanni Federico, Silvia Del Ry

**Affiliations:** 1 Biochemistry and Molecular Biology Laboratory, CNR, Institute of Clinical Physiology, 56100 Pisa, Italy; manuela.cabiati@ifc.cnr.it (M.C.); m.fontanini3@studenti.unipi.it (M.F.); m.giacomarra@studenti.unipi.it (M.G.); 2 Department of Control and Computer Engineering, Politecnico di Torino, 10121 Torino, Italy; gianfranco.politano@polito.it; 3 Unit of Pediatric Endocrinology and Diabetes, Department Clinical and Experimental Medicine, University of Pisa, 56126 Pisa, Italy; emioli.randazzo@gmail.com (E.R.); diego.peroni@unipi.it (D.P.); giovanni.federico@med.unipi.it (G.F.)

**Keywords:** childhood obesity, biomarkers, LncRNAs, miRNAs, Real-Time PCR

## Abstract

**Background and Methods:** Long non-coding RNAs (LncRNAs) and microRNAs are involved in the pathogenesis of obesity, a multifactorial disease that is characterized by inflammation, cardiometabolic complications, and increased cancer risk among other co-morbidities. The up/down regulation of LncRNAs and microRNAs may play an important role in this condition to identify new diagnostic/prognostic markers. The aim of the study was to identify circulating inflammatory LncRNAs in obese adolescents (*n* = 54) and to evaluate whether their expression behaved differently compared to normal-weight adolescents (*n* = 26). To have a more complete insight, the expression of some circulating miRNAs that are linked to obesity (*miR-33a, miR-223, miR-142, miR-199a, miR-181a, and miR-4454*) were also analyzed. **Results:** LncRNAs and miRNAs were extracted simultaneously from plasma samples and amplified by Real-Time PCR. Among the 86 LncRNAs that were analyzed with custom pre-designed plates, only four (*RP11-347E10.1, RP11-10K16.1, LINC00657, and SNHG12*) were amplified in both normal-weight and obese adolescents and only SNHG12 showed significantly lower expression compared to the normal-weight adolescents (*p* = 0.026). Circulating miRNAs showed a tendency to increase in obese subjects, except for *miR-181a* expression. LncRNAs and miRNAs correlated with some clinical and metabolic parameters. **Conclusions:** Our results suggest the importance of these new biomarkers to better understand the molecular mechanisms of childhood obesity and its metabolic disorder.

## 1. Introduction

Obesity is one of the most common pathological conditions in the world, not only in adults but also in the pediatric population. Obese children/adolescents frequently have metabolic and non-metabolic risk factors that are associated with various complications. The probability of acquiring traits such as dyslipidemia, impaired glucose metabolism, and hypertension is much greater than in the adult obese subject, increasing the risk of developing cardiovascular and metabolic diseases [1,2,3]. It has also been reported that there is an association between obesity and increased cancer risk also in childhood obesity [4,5]. The prevalence of childhood obesity is raised by incorrect lifestyles, suggested by the environment surrounding the individual. Also, the family context plays an important role, mainly due to the socio-economic development that took place in the last century. Therefore, prevention, especially in children, has been considered the best approach to decrease the prevalence of obesity. Thus, the measurement of biomarkers that reflect the underlying biological mechanisms of increased disease risk may be an alternative approach to characterize the relevant obesity phenotype. Biomarkers are measurable and quantifiable biological parameters that can be used as indexes to evaluate a biological state: studies in the literature concerning biomarkers in the field of childhood obesity included, so far, adipokines, cytokines, and metabolites [6,7,8,9]. Moreover, it was recently observed that there was a presence of significant levels of nuclear damage in peripheral cells of obese and overweight children that can be not completely repaired, causing chromosome alterations. Increased basal metabolism together with a low-grade inflammation lead, and successively amplify, oxidative stress, stimulating the production of molecules compromising the DNA integrity of peripheral cells [5]. In recent years, the search for new potential biomarkers has also been directed to non-coding RNAs; while for microRNAs (miRNAs) there is specific evidence in the literature of their role as biomarkers [10], data regarding long non-coding RNAs (LncRNAs) are few in the field of childhood obesity. LncRNAs represent a heterogeneous class of non-coding RNAs with a length that is greater than 200 nucleotides, that regulate gene expression both at the transcriptional and post-translational level, in different physiological and/or pathological biological processes [11,12,13,14]. Finally, LncRNAs seem to be involved in the pathogenesis of obesity and their up/down-regulation may play an important role in this condition [15,16]. As above reported, obesity is a multifactorial disease that is characterized by inflammation leading to vascular and cardiometabolic alterations among other co-morbidities. Thus, an interesting point would be to recognize not only the possible link between LncRNAs and obesity, but also to analyze whether changes in the LncRNAs expression may have a role in the obesity-associated inflammation. Some studies that were performed in obese adults showed that some LncRNAs are linked to the obesity-associated chronic inflammatory disease, while others to insulin resistance and type 2 diabetes mellitus [17,18,19]. Obese subjects show increased circulating values of cytokines and adipokines, representing a marker of inflammation and a risk factor for cardiometabolic co-morbidities [18,19]. As in the pediatric population, a long-lasting inflammation is relevant to the evolution of obesity-related co-morbidities, the purpose of this study was to identify, among a large set of circulating LncRNAs that are linked to inflammation, those that are differentially expressed in obese and normal-weight adolescents. Furthermore, to analyze in more detail the biomolecular profile of the subjects that were enrolled and to better describe the inflammatory process, we also investigated the expression levels of some circulating miRNAs that are known to be involved in obesity.

## 2. Materials and Methods

### 2.1. Subjects

The study protocol was approved by the Local Ethics Committee of the Azienda Ospedaliero-Universitaria Pisana (AOUP) and conducted according to the principles of the Declaration of Helsinki for clinical trials. Informed consent was obtained from the parents and from each subject as appropriate.

Thanks to the collaboration with the AOUP Unit of Pediatric Endocrinology and Diabetes (Department of Clinical and Experimental Medicine, University of Pisa, Italy), we enrolled 80 adolescents, 54 obese (O) (12.5 ± 0.41 years) that were not suffering from cardiovascular diseases and 26 sex- and age-matched normal-weight (N) subjects. The normal-weight adolescents were healthy subjects and showed normal biohumoral parameters. The obese adolescents had primary obesity that was not induced by drugs or other pathologic conditions, and none of them had diabetes or cardiac dysfunction.

Specific reference values for age and sex were used for the definition and estimation of the z-scores for weight and height [20]; in detail, the body mass index (BMI) was calculated using the formula weight [(Kg)]/height [(m)^2^] [21]. Body composition was measured with a segmental body composition analyzer (Tanita BC-418 Segmental Body Composition Analyzer, Tanita Corporation, Tokyo, Japan) [22], and the blood pressure was measured in agreement to the standardized protocol [23]. The blood samples were collected in the morning, after overnight fasting, in tubes containing EDTA (1 mg/mL) and aprotinin (500 KIU/mL mL) to inhibit coagulation and to prevent proteolysis. The circulating levels of glucose and lipids were measured after collecting the blood samples in propylene tubes containing lithium-heparin, while to assay those of insulin, the samples were collected in tubes containing EDTA (Vacutest, Kima Srl, Arzergrande, PD, Italy). The measurements of blood glucose, total cholesterol, HDL, LDL, and triglycerides were carried out using automated enzymatic methods (Cobas Integra 400 analyzer, Roche, Italy). Insulin was measured by a standardized commercial immunoassay (Access^®^ Ultrasensitive Insulin, Beckman Coulter Inc., Fullerton, CA, USA), with a sensitivity of 0.03 μIU/mL and precision <10%. The HOmeostasis Model Assessment of Insulin Resistance (HOMA-IR) index was calculated using the formula: fasting plasma insulin [μU/mL] x fasting plasma glucose [mmol/L]/22.5. The serum C-reactive protein (CRP) levels were measured by high sensitivity turbidimetric method (Kamiya Biomedical Co., Seattle, WA, USA) with a sensitivity of 0.005 mg/dL and inter- and intra-assay variability <4%. The endothelial function was evaluated by measuring the reactive hyperemia index (RHI) at the peripheral level with a non-invasive method using the Endo-PAT2000 (Itamar Medical Ltd., Cesarea, Israel) [24,25].

### 2.2. RNA Extraction

Circulating LncRNAs and miRNAs were extracted simultaneously from adolescent plasma samples using the phenol-guanidine thiocyanate method (QIAZOL Lysis Reagent & miRNeasy) with a dedicated kit (miRNeasy Serum/Plasma kit, Qiagen, Hilden, Germany). Briefly, 200 μL of plasma were lysed in adequate lysis reagent releasing the nucleic acids in solution as well as inactivating the endogenous RNase. The samples were then applied to silica-membrane columns that bind total RNA, allowing phenol and other contaminants to be efficiently washed away. Specifically, for plasma miRNA expression profiling, we used an exogenous control, called miRNA mimic, derived from *C. elegans* (Syn-cel-miR-39 miScript miRNA Mimic, Spike-In) to help monitoring RNA recovery and reverse transcription efficiency. This miRNA mimic can be easily detected by Real-Time PCR using the miScript PCR System in combination with the Ce_miR-39_1 miScript Primer Assay. High-quality RNA was then eluted in 14 μL of RNase-free water. All the samples were stored at −80 °C until used after integrity, purity, and concentration evaluation.

### 2.3. miRNAs and LncRNAs Reverse Transcription and Real-Time PCR

The fraction of mature miRNA that was present in the total RNA was reverse-transcribed using the miScript II RT kit (Qiagen), starting from 1 μg/sample in 20 μL of final reaction volume. As LncRNAs circulated in the blood at low concentrations, a pre-amplification reaction was required. It was carried out with a dedicated kit (iScript Explore One-Step RT and PreAmp Kit, Bio-Rad, Hercules, CA, USA) that was able to produce pre-amplified cDNA directly from a small amount of extracted RNA (10.5 μL), combining pre-amplification and reverse transcription in a single reaction. Real-Time PCR analyses were performed in a Bio-Rad C1000™ thermal cycler (CFX-96 Real-Time PCR detection systems, Bio-Rad Laboratories Inc., Hercules, CA, USA). To investigate the expression of miRNAs, we employed a classic 96 well Real-Time PCR plate, after designing the primers with the miRBase database (www.mirbase.org, (accessed on 23 December 2021) Table 1) To analyze the expression of LncRNAs we used a commercially-produced custom pre-designed plate (PrimePCR™ PCR, Assays, Bio-Rad Laboratories Inc., Hercules, CA, USA) in which 86 highly specific inflammatory LncRNA primers (chosen by the manufacturer through the use of bioinformatics tools for inflammatory pathways), housekeeping genes (Beta-2-Microglobulin B2M, Hydroxymethylbilane Synthase HMBS, TATA-Box Binding Protein, TBP), and PCR efficiency controls were already spotted into the wells. The miRNA- and LncRNA-specific reaction mixtures were then generated and SYBR Green (SsoFAST EvaGreen Supermix and SsoAdvanced ™ Universal SYBR^®^ Green Supermix) was used to monitor cDNA amplification. In order to verify the specificity of the amplified products, the amplicons were tested by the analysis of the melting curves, generated from +65 °C to +95 °C with an increase of 0.5 degrees at each cycle.

### 2.4. Statistics

The results from Real-Time PCR of LncRNAs were normalized with a set of 3 stable reference genes (B2M, HMBS, TBP) that were carefully selected by the manufacturer of the custom pre-designed plates, whose stability was estimated through the combination of GeNorm technology and CFX Maestro Manager Software (Bio-Rad, Laboratories Inc., Hercules, CA, USA). MiRNA mimic Ce_miR-39, supplied by Qiagen as housekeeping gene, was used to normalize the miRNA expression data. The relative quantification of the results was carried out with the 2-∆∆Cq method [26].

The statistical analysis was carried out using Stat-View 5.0.1 software (1992–98, SAS Institute Inc., SAS campus Drive, Cary, NC, USA). We used the Kolmogorov–Smirnov test of normality to verify whether the variables had a Gaussian distribution. According to the level of distribution, the data are presented as the mean ± SEM. The skewed variables were logarithmically transformed before analysis. The unpaired Student’s *t*-test was used to compare the normally distributed variables and the logarithmically-transformed variables. A Fisher’s test was used for multiple comparisons, after analysis of variance (ANOVA). The correlations were evaluated by linear regression analysis. A two-tailed *p*-value < 0.05 was considered as statistically significant.

The clustergram graph was built using CFX Maestro Manager Software (Bio-Rad, Laboratories Inc, Hercules, CA, USA); it shows the data in a hierarchy based on the degree of similarity of expression for different targets and samples.

In addition, a dynamic heat-map of the expression of LncRNAs was also generated by specific tools of bioinformatics at the Polytechnic University of Turin.

## 3. Results

O and N adolescents showed different clinical and biochemical characteristics, as reported in Table 2A.

For the O patients, both males and females, were taller than their N counterparts, as shown by their higher height z-score. As expected, the O subjects had higher weight, BMI, waist circumference, and fat mass (%) compared to the N subjects. As blood pressure changes according to height, age, and sex, we analyzed it separately in males and females and we observed that the obese females had significantly higher blood pressure levels than the normal-weight ones. Such a difference was not observed in the males. The O patients had also a significantly slight increase in pubertal development as a consequence of their nutritional excess. With regards to their biochemical characteristics, the O subjects showed a significant increase in the circulating levels of glucose, insulin, total, and LDL cholesterol in comparison with the N subjects and also the HOMA-IR values were higher in the O subjects than in the N subjects. The plasma levels of CRP, an index of inflammation, were also increased in the O subjects than in the N subjects.

The analysis of the pre-designed Real-Time PCR plates, initially carried out in a subgroup of obese and control subjects (O = 10, N = 7, see clinical details in Table 2B), showed that not all the 86 LncRNAs were expressed in all the samples and in the same way.

**Figure 1:**
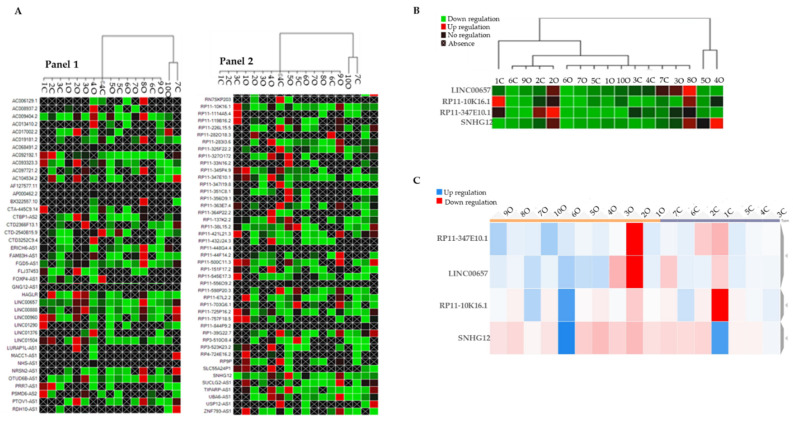
(**A**) Clustergram graph: LncRNAs expression in obese in comparison with the mean expression in normal-weight adolescents. Red square: up-regulation, green square: down-regulation, black square: no regulation; black crossed square: absence of expression. Panel 1 reports 42 LncRNA and Panel 2 the remaining 45 LncRNAs for a total of 86 LncRNAs that were analyzed. (**B**) Clustergram of the four selected inflammatory LncRNAs (*LINC00657, RP11-10K16.1, RP11-347E10.2, and SNHG12*). On the columns the acronyms of the obese and normal-weight subjects, on the lines the names of the LncRNAs; green represents down-regulation, red represents up-regulation. (**C**) Dynamic heat-map graph of *LINC00657, RP11-10K16.1, RP11-347E10.2*, and *SNHG12* in the obese and control group. On the column are the number of obese and normal-weight samples. Red square: down-regulation, blue square: up-regulation.

By using the CFX Maestro Software (Bio-Rad, Laboratories Inc, Hercules, CA, USA), we generated a clustergram graph based on the comparison between the mean expression of LncRNAs that were observed in the O subjects in comparison with the N subjects (Figure 1A). The clustergram depicts the relative degree of expression by a scale of colors: the up-regulation (higher expression) by a red square; the down-regulation (lower expression) by a green/blue square; and no regulation by a black square. The lighter the shade of color was, the greater the relative expression difference was. The criterion to select LncRNAs was to identify those that were expressed in samples from all the O subjects and the N subjects. This criterion was fulfilled *by LINC00657, RP11-10K16.1, RP11-347E10.2*, and *SNHG12* (Figure 1B).

To better visualize their trend, we reported the expression values of the four identified LncRNAs in a dynamic heat-map graph (Figure 1C). This allowed a visual representation of the adjustment of the targets for an experimental sample compared with a control sample that was based on the normalized relative expression and its position on the plate. The heat-map of the results of this work is available at https://orion.polito.it/other-websites/heatmapper/ (accessed on 23 December 2021).

By placing the O subjects and the N subjects in two different groups exploiting the bioinformatic parameters, we observed that, while in the obese group the expression of the LncRNAs was tendentially homogeneous compared with the normal-weight group, *SNHG12* showed a uniform pattern of color in both the groups.

**Figure 2:**
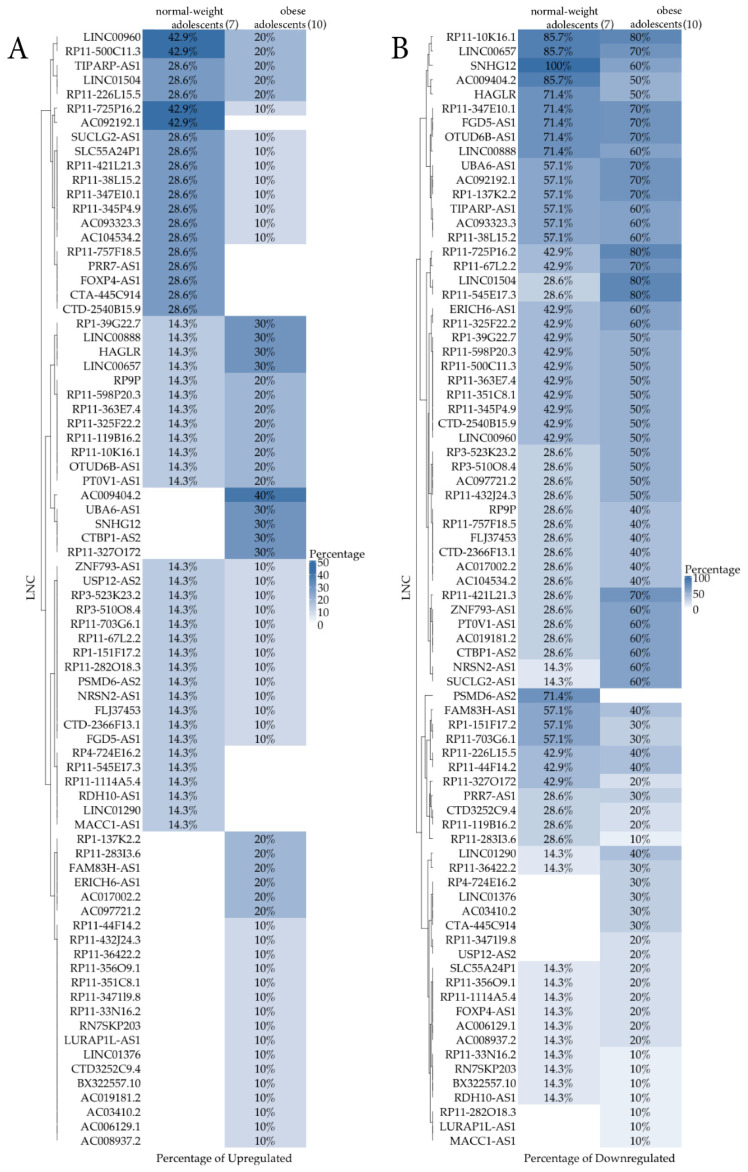
Heatmap comparing the percentage of samples that were expressing (**A**, up-regulation)/not expressing (**B**, down-regulation) LncRNA out of 7 normal-weight and 10 obese subjects.

A heatmap representation (Figure 2) per group comparing the percentage of samples that were expressing (Figure 2A)/not expressing (Figure 2B) LncRNAs out of 7 normal-weight and 10 obese subjects was also reported. The Figure 2A shows the percentage of up-regulated LncRNAs, whereas the Figure 2B shows the down-regulated ones. While the first graph is not very relevant due to the low differential percentages that were obtained, the second one appears more informative. From this qualitative analysis, a new group of nine LncRNAs (*RP4-724E16.2, LINC01376, AC03410.2, CTA-445C914, RP11-347119.8, USP12-AS2, RP11-282O18.3, LURAP1L-AS1*, and *MAK1-AS1*), not shown by the previous quantitative analysis, were identified. They could have an interesting role in childhood obesity to be clarified in future studies.

**Figure 3:**
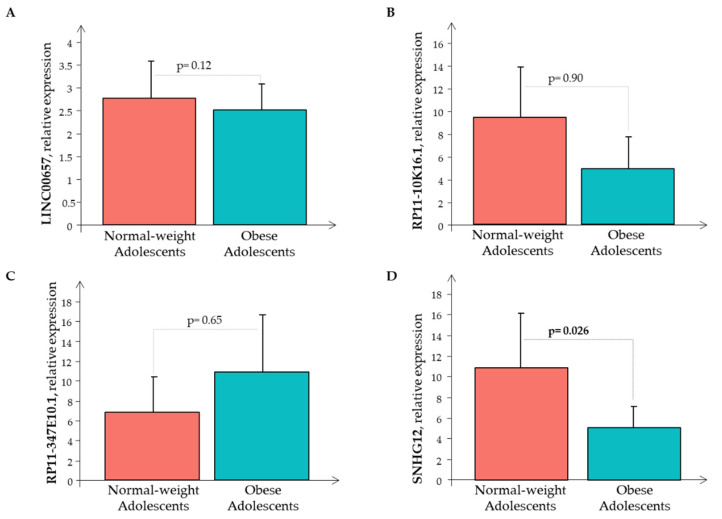
The relative expression of LncRNAs (**A**) *LINC00657*, (**B**) *RP11-10K16.1*, (**C**) *RP11-347E10.1*, and (**D**) *SNHG12* in the normal-weight (red bar) and the obese (cyan bar) subjects.

Subsequently, the Real-Time PCR analysis of the four LncRNAs was performed in all the 80 subjects showing that only *RP11-347E10.1* had higher expression levels in the O subjects than in the N subjects, while the remaining circulating LncRNAs had decreased expression levels in the O subjects than in the N subjects, that was significant only for *LncRNA SNHG12* (Figure 3).

**Figure 4:**
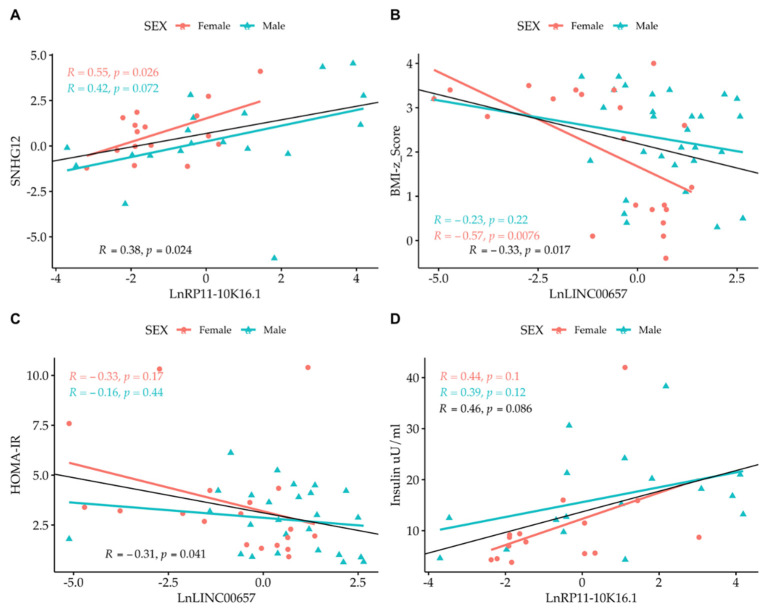
Simple regression analysis: (**A**) *Ln SNHG12* vs. *Ln RP11-10K16.1*; (**B**) *LINC00657* vs. BMI-z score; (**C**) *LINC00657* vs. HOMA-IR; and (**D**) *RP11-10K16.1* vs. insulin split by gender. Light red circle: female; cyan triangle: male.

**Figure 5:**
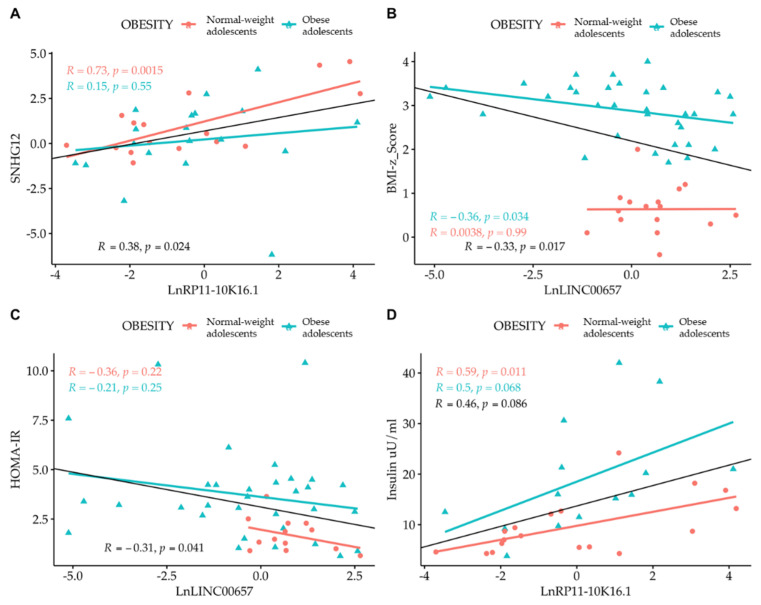
Simple regression analysis: (**A**) *Ln SNHG12* vs. *Ln RP11-10K16.1*; (**B**) *LINC00657* vs. BMI-z score; (**C**) *LINC00657* vs. HOMA-IR; and (**D**) *RP11-10K16.1* vs. insulin split by obesity. Light red circle: normal-weight adolescents; cyan triangle: obese adolescents.

Correlation analysis among the LncRNAs themselves showed that only *SNHG12* and *RP11-10K.16.1* were correlated to one another. By correlating the expression of LncRNA with the values of clinical parameters, we observed the presence of an inverse correlation between *LncRNA LINC00657* and the BMI-z score and HOMA-IR, and a direct correlation between *LncRNA RP11-10K16.1* and insulin (Figure 4 and Figure 5). The regression analyses were also performed according to gender (Figure 4, panel A–D) and separately in normal- weight and obese subjects. (Figure 5, panel A–D).

A strong correlation between *LINC00657*and CRP in females (R = 0.53, *p* = 0.016) was also observed.

**Figure 6:**
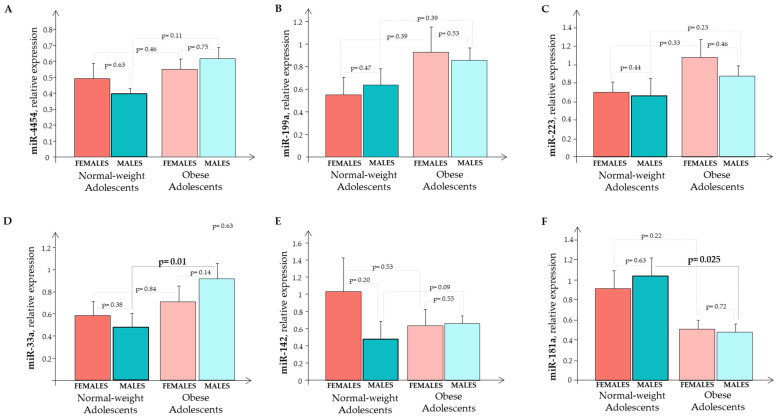
The relative expression of circulating miRNA (**A***) miR-445*; (**B**) *miR-199a*; (**C**) *miR-223*; (**D**) *miR-33a*; (**E**) *miR-142*; and (**F**) *miR-181a* in normal-weight and obese subjects that were split by gender. Normal-weight females (red bar); normal-weight males (cyane bar); obese females (light red bar); obese males (light cyane bar).

The expression of circulating *miR-33a, miR-223, miR-142, miR-199a, miR-4454*, and *miR-181a* in the O subjects and the N subjects were evaluated according to gender (Figure 6). We observed a significant difference for *miR-33a* and *miR-181a* in the males (Figure 6D,F).

Analyzing the whole sample (males + females), we observed a significant, higher expression in *miR-33a* (*p* = 0.039) and *miR-181a* (*p* = 0.011) in the O subjects than in the N subjects.

We performed a regression analysis to detect the potential relationships between miRNAs and LncRNAs. While we did not find any relationship between the circulating miRNAs and LncRNAs, we observed, when analysing the whole enrolled population (O and N subjects), the presence of significant correlations among the circulating miRNAs (Table 3) and between the miRNAs and parameters such as BMI, BMI z-score, waist circumference, blood insulin, and CRP levels (Table 4). By performing the same correlations separately in the O and the N subjects, we observed that a significant relationship among the miRNAs was still present in the obese individuals with the exception of miR-181a vs. miR-199a and miR-4454 (Table 3). With regards to the relationships between the miRNAs and clinical and biochemical variables, we found a significant correlation in obese subjects for BMI z-score vs. miR-4454, miR-199a, miR-223 and miR-33a, while BMI and WC correlated with miR-4454 (Table 4).

## 4. Discussion

Obesity is one of the most widespread pathological conditions in the world, with a steady increase in recent decades and with a high prevalence in children. The clinical features of an obese adolescent are characterized by metabolic and non-metabolic risk factors that are related to various co-morbidities, starting from cardiovascular diseases up to cancer [3,27].

In this regard, it was interesting to observe that our obese subjects, when compared to normal-weight counterparts, already had some metabolic alterations such as a slight increase in blood glucose and an increase in the circulating insulin levels and in HOMA-IR, suggesting that a certain degree of insulin resistance was operative despite their young age. Also, the plasma levels of total cholesterol and of the LDL fraction were increased underlying the presence of an altered lipid metabolism. The higher circulating CRP levels suggested that the obese subjects had a low-grade inflammation. Looking at the blood pressure, we observed that both systolic and diastolic blood pressures were significantly higher in the obese females, but not in the males, in comparison with their counterparts. This finding may, in part, reflect the higher level of obesity in females in comparison with males (BMI z-score: females 3.1 ± 0.1 vs. males 2.7 ± 0.1; *p =* 0.018) and a slightly higher stage in sexual development in obese females than in males, even if it was not significant (Tanner stage: obese females 4.1 ± 0.2 vs. obese males 3.5 ± 0.1; *p =* ns). Previous studies indicated that obesity per se is an independent predictor of some adverse cardiovascular changes [28] and that sexual maturation, through changes in estrogen, progesterone, and testosterone, may influence arterial wall properties and function [29]. In a previous study from our group, we observed that obese children and adolescents had structural and functional alterations of the heart and large vessels reflecting both physiologic adaptations to an increase in body size (in free fat mass in particular) and adverse effects of metabolically active intra-abdominal adiposity [30]. Some previous data suggested that these changes are fully reversible with weight loss [31]. In the present study, however, obese and normal-weight subjects had similar RHI values, indicating that the endothelial function was still conserved in our obese individuals. Therefore, a non-invasive, early assessment and monitoring of obese/overweight children and adolescents may be useful to prevent/mitigate obesity and cardiovascular complications in adulthood.

In this regard, in the last years, the search for early diagnostic/prognostic biomarkers in childhood obesity played an important role in the prevention and treatment of the disease: in addition to the more classic ones [5,8,32,33], recently the interest was directed to the non-coding portion of RNA to detect possible biomarkers among the non-coding RNAs [34]. While there is evidence from the literature for a role of miRNAs in obesity [35], data concerning the expression of circulating LncRNAs are few in childhood obesity.

We started the search for circulating LncRNAs that were possibly involved in childhood obesity from the known pro-inflammatory state that is typical of obese subjects [36], thus 86 inflammatory LncRNAs were selected, evaluating their transcriptional level variations. From this large set of LncRNAs, four, that were expressed in all the samples under examination, were chosen: *LINC00657, RP11-347E10.1, RP11-10K16.1*, and *SNHG12*. Studies in the literature showed that they are involved in various pathological conditions, in particular in carcinogenic processes where the inflammatory component plays an important role. In detail, *LINC00657* is an intergenic LncRNA playing a role in carcinogenesis as well as in angiogenesis in patients with atherosclerosis [37,38,39,40]. While for LncRNAs *RP11-347E10.1* and *RP11-10K16.1* there are not implications as yet, for *SNHG12* many reports show its role in carcinogenesis of several tumor types; this antisense LncRNA, has been evaluated as a potential biomarker and therapeutic target in tumorigenesis [41]. Several data showed that *SNHG12* functions as a competing endogenous RNA [*ceRNA*] for some miRNAs, such as *miR-119a/b-5p* in hepatocellular carcinoma and *miR-181a* in non-small cell lung cancer, highlighting the interaction between the two families of non-coding RNA [41,42,43]. Our results showed that the O subjects, when compared with the N subjects, had a slight reduction in the *RP11-347E10.1* expression that was significant for *SNHG12.* To date, these LncRNAs had been considered as factors that are involved in carcinogenesis [37,38,39,40,41,42,43] and this is the first study analyzing these LncRNAs in relation to childhood obesity. Our results suggested that the adolescents we examined had some metabolic alterations, a low level of inflammation, but they did not show signs of active carcinogenesis.

The lack of information on the LncRNAs that we analyzed in obese children and adolescents allowed us to merely hypothesize a link between these LncRNA and the metabolic profile of our O subjects, as suggested by the correlations that we observed. The inverse correlation that we found between LncRNA *LINC00657* and BMI z-score suggests that the expression profile of this LncRNA is influenced by weight. This hypothesis is also reinforced by the correlation that we observed between LncRNA *LINC00657* and HOMA-IR, an index of increased insulin resistance, a metabolic alteration that is typical of obese subjects. Furthermore, the positive correlation that we found between the transcriptional levels of LncRNA *RP11-10K16.1* and the circulating values of insulin suggested that this LncRNA could be associated with hyperinsulinism, a common condition that is observed in obese subjects.

A recent study [44] exploring the LncRNA-mRNA co-expression profile in adipose tissue of obese and non-obese children using microarray technology, found a large amount of LncRNAs and mRNAs that are linked to childhood obesity, which were differentially expressed between the two groups. The authors concluded that the differentially expressed LncRNAs interacted with the differentially expressed genes, providing a novel perspective on the mechanisms of childhood obesity, which is conducive to the search for new diagnostic and therapeutic strategies [44]. Furthermore, Cui et al., reported the identification of three circulating miRNAs that were able to predict the future risk of type 2 diabetes mellitus in obese children [45].

Our own results in obese children and adolescents, together with those from other authors [44,45], may integrate those that were found in obese adults, in whom it has been identified a number of LncRNAs that are linked to obesity [45,46,47,48]. In particular, it was observed that some LncRNAs are linked to the obesity-associated chronic inflammatory disease, while others to insulin resistance and type 2 diabetes mellitus [17]. It is interesting to note that LncRNAs that are implicated in mediating a chronic low-grade inflammatory obese state have the potential to be the next generation of biomarkers and therapeutic targets owing to their tissue-specific nature of expression [49]. In obese adults, LncRNAs that are linked to weight loss, which show promising mechanistic relevance, have been also identified [50]. Possibly, in the near future, coupling both lifestyle changes and targeting of LncRNAs in various obesity-associated inflammatory conditions may be of therapeutic benefit.

In line with previous studies [35,51], our data showed that the expression of miRNAs tended to increase in the O subjects compared with the N adolescents, reaching statistical significance only for *miR-33a*, a known regulator of lipid metabolism and vascular homeostasis, confirming their involvement in obesity [52,53,54]. In addition, *miR-181a* was significantly lower in the O subjects than in the N subjects. This result is consistent with the literature reports showing that a reduced *miR-181a* expression level in obese patients is an index of the onset of metabolic disorders and cardiovascular disease [55]. Correlation analyses showed positive relationships among the circulating miRNAs themselves and between the miRNAs and clinical parameters such as the BMI z-score, fat mass, and waist circumference, confirming their involvement in obesity. In this regard, it is interesting to note that we observed the presence of correlations between *miR-4454, miR-199a, miR-223, miR-33a*, and BMI z-score, BMI and WC in obese individuals even when the data were analyzed separately in obese and normal-weight subjects. In addition, the absence of correlations between LncRNA *SNHG12* and *miR-199a* and *miR-181a*, that are known to be associated to carcinogenesis, supports the concept that in our O subjects, a process of active carcinogenesis was not operative.

## 5. Conclusions

If correct in our assumption, then our data suggest that our O subjects, having primary obesity without associated complications, show signatures indicating that they are going to develop metabolic alterations. This aspect is of overwhelming interest if one considers that the recognition of specific LncRNA that are linked to obesity may be helpful to identify, very early, subjects that are at higher risk of obesity and its cardiometabolic complications that, being at an initial stage, might be mitigated/reverted by lifestyle changes. Furthermore, in the near future, by determining the mode of action of LncRNAs and understanding their role in regulating obesity-related inflammatory processes, LncRNAs-mediated targets that are suitable for therapeutic intervention could be identified.

However, the precise mechanisms through which non-coding RNAs are involved in obesity and its co-morbidities, and/or in predicting their progression remain to be clarified. This study represents a first step in investigating the implication of lncRNAs in childhood obesity and further studies on a larger number of samples are needed to disclose the role of non-coding RNAs in this condition and related co-morbidities.

## Figures and Tables

**Table 1:** Mature miRNA sequences.

Gene	Forward Primer Sequence (5′-3′)	Genbank Accession Number	Location	Temperature °C
*hsa-miR-33a-3p*	CAATGTTTCCACAGTGCATCAC	NR_029507	chr 22 q13.2	55
*hsa-miR-223-5p*	CGTGTATTTGACAAGCTGAGTT	LM608368	chr X q12	55
*hsa-miR-142-5p*	CATAAAGTAGAAAGCACTACT	NR_029683	chr 17 q22	55
*hsa-miR-199a-5p*	CCCAGTGTTCAGACTACCTGTTC	NR_029586	chr 19 p13.2	55
*hsa-miR-4454*	GGATCCGAGTCACGGCACCA	NR_039659	chr 4 q32.2	55
*hsa-miR-181a-5p*	AACATTCAACGCTGTCGGTGAGT	NC_000009.12	chr 22 1q32.1	55

Table Legend. *hsa-miR-33a-3p*: homo sapiens *microRNA-33a* with 3p strand present in the reverse position; *hsa-miR-223-5p*: homo sapiens *microRNA-223* with 5p strand present in the forward position; *hsa-miR-142-5p*: homo sapiens *microRNA-142* with 5p strand present in the forward position; *hsa-miR-199a-5p*: homo sapiens *microRNA-199a* with 5p strand present in the forward position; *hsa-miR-4454*: homo sapiens *microRNA-4454*; *hsa-miR-181a-5p*: homo sapiens *microRNA-181a* with 5p strand present in the forward position.

**Table 2:** (**A**) Clinical characteristics, body composition, and biochemical variables of the study population. (**B**) Clinical characteristics of the subjects that were included in the subgroups.

(A)			
	Normal-Weight Subjects (*n* = 26)	Obese Subjects (*n* = 54)	*p*
*Clinical parameters*			
Age (years)	12.8 ± 0.2	12.5 ± 0.41	n.s.
Male: Female	14:12	34:20	n.s.
Pubertal Stage	3.2 ± 0.2	3.7 ± 0.1	n.s.
Height z-score	0.3 ± 0.15	0.9 ± 0.1	<0.001
Weight (Kg)	52.6 ± 2.4	74.3 ± 2.1	<0.0001
BMI	21.1 ±0.54	29.4 ± 0.65	<0.0001
BMI z-score	0.7 ± 0.05	2.8 ± 0.06	<0.0001
FM (%)	18 ± 1.0	36 ± 1.0	<0.0001
WC (cm)	76.0 ± 0.4	94.8 ± 2.0	<0.0001
WC/H	0.48 ± 0.004	0.61 ± 0.001	<0.0001
SBP (mmHg) females	106 ± 1.8	113 ± 2.3	<0.03
DBP (mmHg) females	61 ± 1.7	67 ± 1.4	<0.01
SBP (mmHg) males	116 ± 0.9	111 ± 2.0	n.s.
DBP (mmHg) males	62 ± 1.9	63 ± 1.3	n.s.
***Hematochemical parameters***			
Glycemia (mg/dL)	77 ± 1.5	87 ± 1.6	<0.0001
Insulin (uU/mL)	7.3 ± 0.4	19.2 ± 1.5	<0.0001
Homa-IR	1.5 ± 0.1	3.7 ± 0.3	<0.0001
Cholesterol (mg/dL)	133 ± 8.8	169 ± 4.1	<0.0001
HDL (mg/dL)	45 ± 3.0	48 ± 1.4	n.s.
LDL (mg/dL)	73 ± 6.3	104 ± 3.8	<0.0001
Triglycerides (mg/dL)	69 ± 15	86 ± 6.7	n.s.
CRP (mg/dL)	0.04 ± 0.02	0.33 ± 0.01	<0.0001
RHI	2.1 ± 0.05	1.4 ± 0.05	<0.0001
**(B)**			
	**Normal-Weight Subjects** **(*n* = 7)**	**Obese Subjects** **(*n* = 10)**	***p***
***Clinical parameters***			
Age (years)	12.9 ± 0.2	12.2 ± 0.3	n.s.
Male: Female	5:4	3:5	n.s.
Pubertal Stage	3.3 ± 0.3	3.7 ± 0.4	n.s.
Height z-score	0.2 ± 0.1	1.0 ± 0.2	0.03
Weight (Kg)	50.4 ± 1.5	65.3 ± 4.2	<0.001
BMI	20.2 ±0.4	29.1 ± 1.2	<0.0001
BMI z-score	0.6 ± 0.1	2.8 ± 0.2	<0.0001
FM (%)	18.3 ± 1.8	38.2 ± 2.4	<0.0001
WC (cm)	76.4 ± 0.5	91.5 ± 3.3	<0.002
WC/H	0.48 ± 0.006	0.63 ± 0.01	<0.0001
SBP (mmHg) females	107 ± 2.2	114 ± 1.1	<0.03
DBP (mmHg) females	60 ± 1.3	66 ± 0.6	<0.007
SBP (mmHg) males	115 ± 1.4	112 ± 0.8	n.s.
DBP (mmHg) males	61 ± 1.3	62 ± 1.5	n.s.

The data are expressed as the mean ± SEM. **Table Legend.** BMI: body mass index; FM: fat mass; WC: waist circumference; WC/H: ratio waist circumference and height; SBP: sistolic blood pressure; DBP: diastolic blood pressure; HOMA-IR: Homeostasis model assessment of insulin resistance; HDL: high density lipoprotein; LDL: low density lipoprotein; CRP: C-reactive protein; RHI: reactive hyperemia index.

**Table 3:** Correlations among the circulating miRNAs.

	*miR-33a* *miR-33a N/O*	*miR-223* *miR-223 N/O*	*miR-142* *miR-142 N/O*	*miR-199a* *miR-199a N/O*	*miR-4454* *miR-4454 N/O*	*miR-181a* *miR-181a N/O*
***miR-33a*** ***miR-33a*** **N** ***miR-33a*** **O**	-	R = 0.36; *p* = 0.002 R = 0.17; *p* = 0.44 R = 0.38; *p* = 0.006	R = 0.40; *p* = 0.0008 R = 0.30; *p* = ns R = 0.45; *p* = 0.001	R = 0.34; *p* = 0.005 R = 0.08; *p* = ns R = 0.46; *p* = 0.001	R = 0.30; *p* = 0.01 R = 0.22; *p* = ns R = 0.29; *p* = 0.037	-
***miR-223*** ***miR-223*** **N** ***miR-223*** **O**	R = 0.36; *p =* 0.002 R = 0.17; *p =* 0.04 R = 0.38; *p =* 0.006	-	R = 0.53; *p*< 0.0001 R = 0.64; *p =* 0.002 R = 0.49; *p =* 0.0003	R = 0.27; *p =* 0.03 R = 0.02; *p =* ns R = 0.30; *p =* 0.04	R = 0.53; *p* < 0.0001 R = 0.21; *p =* ns R = 0.60; *p* < 0.0001	-
***miR-142*** ***miR-142*** **N** ***miR-142*** **O**	R = 0.40; *p =* 0.0008 R = 0.30; *p =* ns R = 0.45; *p =* 0.001	R = 0.53; *p*< 0.0001 R = 0.64; *p =* 0.002 R = 0.49; *p =* 0.0003	-	R = 0.61; *p*< 0.0001 R = 0.62; *p =* 0.004 R = 0.59; *p* < 0.0001	R = 0.58; *p*< 0.0001 R = 0.54; *p =* 0.012 R = 0.60; *p* < 0.0001	-
***miR-199a*** ***miR-199a*** **N** ***miR-199a*** **O**	R = 0.34; *p =* 0.005 R = 0.08; *p =* ns R = 0.46; *p =* 0.001	R = 0.27; *p =* 0.03 R = 0.02; *p =* ns R = 0.30; *p =* 0.04	R = 0.61; *p* < 0.0001 R = 0.62; *p =* 0.004 R = 0.59; *p* < 0.0001	-	R = 0.45; *p =* 0.0001 R = 0.50; *p =* 0.022 R = 0.41; *p =* 0.004	R = 0.30; *p =* 0.02 R = 0.70; *p* < 0.0001 R = 0.23; *p =* ns
***miR-4454*** ***miR-4454*** **N** ***miR-4454*** **O**	R = 0.30; *p =* 0.01 R = 0.22; *p =* ns R = 0.29; *p =* 0.037	R = 0.53; *p* < 0.0001 R = 0.21; *p =* ns R = 0.60; *p* < 0.0001	R = 0.58; *p*< 0.0001 R = 0.54; *p =* 0.012 R = 0.60; *p* < 0.0001	R = 0.45; *p =* 0.0001 R = 0.50; *p =* 0.022 R = 0.41; *p =* 0.004	-	R = 0.24; *p =* 0.05 R = 0.60; *p =* 0.03 R = 0.18; *p =* ns
***miR-181a*** ***miR-181a*** **N** ***miR-181a*** **O**	-	-	-	R = 0.30; *p =* 0.02 R = 0.70; *p =* <0.0001 R = 0.23; *p =* ns	R = 0.24; *p =* 0.05 R = 0.60; *p =* 0.03 R = 0.18; *p =* ns	-

**Table 4:** Correlations between the circulating miRNAs and the clinical parameters.

	Weight (Kg) N/O	BMI z-Score N/O	BMI N/O	WC (cm) N/O	Insulin (mU/mL) N/O	CRP (mg/dL) N/O
***miR-4454*** ***miR-4454* N** ***miR-4454*** **O**	R = 0.36; *p =* 0.02 R = 0.38; *p =* ns R = 0.37; *p =* ns	R = 0.40; *p =* 0.0006 R = 0.03; *p* = ns R = 0.48; *p* < 0.001	R = 0.38; *p =* 0.02 R = 0.37; *p =* ns R = 0.40; *p =* 0.05	R = 0.33; *p =* 0.04 R = 0.23; *p =* ns R = 0.39; *p =* 0.05	-	-
***miR-199a*** ***miR-199a* N** ***miR-199a* O**	-	R = 0.26; *p =* 0.03 R = 0.03; *p =* ns R = 0.29; *p =* 0.05	-	-	-	-
***miR-223*** ***miR-223* N** ***miR-223* O**	-	R = 0.40; *p =* 0.0005 R = 0.49; *p =* 0.02 R = 0.42; *p =* 0.003	-	-	-	R = 0.30; *p =* 0.01 R = 0.51; *p =* 0.014 R = 0.14; *p =* ns
***miR-33a*** ***miR-33a* N** ***miR-33a* O**	-	R = 0.35; *p* = 0.003 R = 0.02; *p =* ns R = 0.34; *p =* 0.02	-	-	R = 0.33; *p =* 0.02 R = 0.37; *p =* ns R = 0.11; *p =* ns	-
***miR-181a*** ***miR-181a* N** ***miR-181a* O**	-	-	-	R = −0.33; *p =* 0.008 R = −0.26; *p =* ns R = 0.22; *p =* ns	-	R = −0.23; *p =* 0.05 R = 0.08; *p =* ns R = 0.009; *p =* ns

## Data Availability

The data that support the findings of this study are available in IFC-CNR.
